# The Diversity of Genetic Outcomes from CRISPR/Cas Gene Editing is Regulated by the Length of the Symmetrical Donor DNA Template

**DOI:** 10.3390/genes11101160

**Published:** 2020-09-30

**Authors:** Amanda M. Hewes, Brett M. Sansbury, Eric B. Kmiec

**Affiliations:** 1 Gene Editing Institute, Helen F. Graham Cancer Center & Research Institute, Christiana Care Health System, Newark, DE 19713, USA; Amanda.M.Hewes@ChristianaCare.org (A.M.H.); sansbury@udel.edu (B.M.S.); 2 Department of Medical and Molecular Sciences, University of Delaware, Newark, DE 19716, USA

**Keywords:** symmetrical homology arms, single-stranded donor template, homology directed repair, CRISPR/Cas12a, CRISPR/Cas9, gene editing

## Abstract

Clustered Regularly Interspaced Short Palindromic Repeats (CRISPR)/Cas gene editing systems have enabled molecular geneticists to manipulate prokaryotic and eukaryotic genomes with greater efficiency and precision. CRISPR/Cas provides adaptive immunity in bacterial cells by degrading invading viral genomes. By democratizing this activity into human cells, it is possible to knock out specific genes to disable their function and repair errors. The latter of these activities requires the participation of a single-stranded donor DNA template that provides the genetic information to execute correction in a process referred to as homology directed repair (HDR). Here, we utilized an established cell-free extract system to determine the influence that the donor DNA template length has on the diversity of products from CRISPR-directed gene editing. This model system enables us to view all outcomes of this reaction and reveals that donor template length can influence the efficiency of the reaction and the categories of error-prone products that accompany it. A careful measurement of the products revealed a category of error-prone events that contained the corrected template along with insertions and deletions (indels). Our data provides foundational information for those whose aim is to translate CRISPR/Cas from bench to bedside.

## 1. Introduction

The emergence of Clustered Regularly Interspaced Short Palindromic Repeats and their associated nucleolytic enzymes (CRISPR/Cas) have revolutionized our capacity to execute genomic engineering in eukaryotic and prokaryotic cells [1,2,3,4,5]. The versatility and ease of use of such systems provides molecular geneticists with an important tool to dissect gene function and the cellular pathways that their expression regulates. CRISPR/Cas can be used to disable functioning genes through the activation of a cellular process known as Non-Homologous End Joining (NHEJ) [2,6]. This DNA damage repair pathway is activated when CRISPR/Cas makes specific double-strand breaks (DSBs) in DNA. As enzymatic processing of the broken DNA ends occurs, single or multiple nucleotides on either or both strands can be lost [6,7]. These changes can result in the alteration of the reading frame and lead to the generation of so-called genetic knockouts through targeted gene editing. 

DSBs can also provide a target for Homology Directed Repair (HDR) activity when a single- or double-stranded template is present [8]. While this can be considered loosely as genetic recombination, the true relationship with this cellular process is that the incoming DNA template must align in homologous register with the target site. The outcome of this process could be the insertion of a short fragment of DNA or the incorporation of a longer fragment which could then serve as a primer for DNA replication [9,10]. These reaction mechanics are not unique to CRISPR-directed gene editing, because they have been reported previously as genetic outcomes in single agent gene editing pathways [11,12,13,14]. Some of these products display a precise insertion or precise correction of the targeted site whereas in other cases, multiple combinations of genetic changes at the DNA level are observed. Recently, Sansbury et al. [15] began to define, catalog, and quantitate the diverse genetic population that resulted from CRISPR-directed gene editing using a mammalian cell-free extract that can support both NHEJ and HDR events simultaneously.

While there is little doubt that the CRISPR/Cas complex is a central component in gene editing reactions, the donor template is responsible for providing the genetic information to catalyze HDR via insertion, nucleotide exchange, or long fragment incorporation. By and large, single-stranded DNA (ssDNA) templates have outperformed double-stranded DNA (dsDNA) templates in HDR reactions and thus have been used more often [8,10,16,17,18,19,20,21,22,23,24,25]. This reaction component plays an important role and can often dictate the degree of success when the goal is to genetically alter a defined section of the genome. 

Several characteristics have been shown to play important roles in the design of donor DNA templates to increase the chances of successful genome editing. Strand bias is one characteristic in which one polarity of the donor DNA, designed to pair a relative strand of the DNA helix, outperforms the other [8,13,14,15,20,26,27,28]. In almost all cases, donor DNA templates that are complementary to the sense strand of the target site catalyze a higher degree of gene editing. Although, strand bias has been shown to be dependent on the genetic sequence within the gene or between genes [5,13,18,29,30]. Another characteristic that can impact genome editing is how the ssDNA donor template is generated. In some cases, this is accomplished through Adeno-Associated viral vectors (AAV) which are introduced into the cell simultaneously with the CRISPR/Cas complex. Alternatively, synthetic DNA oligonucleotides (ODNs) which can also be chemically modified, can increase the binding affinity and enhance stability. Finally, the length of the donor DNA template appears to influence the degree of success in reactions where the gene editing goal is to insert a DNA fragment with precision [5,22,31]. Each of these characteristics is important to the design of donor DNA templates because of the influences they can have on gene editing reactions.

We have been dissecting the mechanism of action and regulatory circuitry surrounding CRISPR-directed gene editing by using a system that employs a cell-free extract and a genetic readout in bacteria. This system enables mechanistic analyses of individual reaction components where each component can be evaluated in a methodical fashion [26]. In addition, gene editing outcomes in this system are not influenced by transfection efficiency of intact cells or the degradation of the CRISPR/Cas complex as it transits to the nucleus [27,28]. This system has already partially elucidated the molecular activity of two CRISPR associated nucleases, Cas12a and Cas9 (CRISPR/Cas12a and CRISPR/Cas9) in both precise and error-prone HDR reaction pathways [15,27]. Our previous studies prompted us to consider the possibility that the length of ssDNA donor templates could also influence the diversity of genetic outcomes in both precise and error-prone gene editing reactions.

In this manuscript, we outline our studies in which we examine the influence of varying lengths of single-stranded donor DNA templates in CRISPR/Cas systems with different programmable nucleases. We chose specific lengths that were sufficiently different while also ensuring robust synthesis and production of full-length, intact molecules. We tested these molecules in reactions catalyzed by two well-known programmable CRISPR nucleases, Cas12a and Cas9. Our results indicate that the degree of precise insertion of a targeted DNA fragment is highly dependent on the length of the donor template in both systems and depending on which nuclease catalyzes the reaction, a diverse array of error-prone HDR products can be seen. 

## 2. Materials and Methods

### 2.1. Preparing Cell-Free Extract

The HEK cell-free extract was prepared in a similar fashion following a method produced by Cole-Strauss, A. et al. [32]. The HEK cell line (American Type Cell Culture, Manassas, VA, USA) was cultured, harvested at 4.5 × 10^6^, and washed with cold hypotonic buffer (20 mM of HEPES, 5 mM of KCl, 1.5 mM of MgCl_2_, 1 mM of dithiothreitol (DTT), and 250 mM of sucrose). Cells were centrifuged based on their respective standard conditions, resuspended in cold hypotonic buffer without sucrose, and incubated on ice for 15 min before being lysed by 25 strokes of a Dounce homogenizer. Cytoplasmic fraction of enriched cell lysate was incubated on ice for 60 min and centrifuged for 15 min at 12,000× *g* at 4 °C. The supernatant was then aliquoted and stored immediately at −80 °C. The concentrations of the cell-free extract were determined using the Bradford assay.

### 2.2. Reaction Conditions

In vitro reactions used an RNP complex containing 10 pmol of purified AsCas12a (AsCpf1) or 10 pmol of SpCas9 with their respective 10 pmol of target-specific crRNA or crRNA+tracrRNA (Integrated DNA Technologies, Coralville, IA, USA). The cleavage reaction mixtures required 500 ng (0.014 μM) of pHSG299 plasmid DNA (Takara Bio Company, Shiga, Japan), 10 pmol of RNP mixed with a reaction buffer (100 mM of NaCl, 20 mM of Tris-HCl, 10 mM of MgCl_2_, and 100 μg/mL of bovine serum albumin), and brought to a final volume of 20 µL. All cleavage reactions were incubated for 15 min at 37 °C and the DNA was recovered and purified using Zymo Research Select-A-Size DNA Clean & Concentrator and Zymo Research DNA Clean & Concentrator-5 kit (Zymo Research, Irvine, CA, USA). The in vitro recircularization reaction contained recovered and purified DNA from the cleavage reaction, approximately 93 µg of cell-free extract supplemented with 400 cohesive end units of Quick T4 Ligase (New England Biolabs, Ipswich, MA, USA), a reaction buffer (20 mM of Tris, 15 mM of MgCl_2_, 0.4 mM of DTT, and 1.0 mM of adenosine triphosphate), and single-stranded donor DNA templates (Integrated DNA Technologies) consisting of symmetrical homology arms of various lengths; 100 pmol of oligonucleotide was added to each recircularization reaction. This secondary reaction was incubated for 15 min at 37 °C then the DNA was recovered and purified once more using both Zymo Research *Select-A-Size* DNA Clean & Concentrator kit and Zymo Research DNA Clean & Concentrator-5 kit (Zymo Research).

### 2.3. Transformation, Selection, DNA Isolation, PCR, and Analysis

Modified plasmid DNA recovered from the in vitro reactions was transformed into 100 μL of Mix & Go!™ Cells, DH5α competent *Escherichia coli*, (Zymo Research) incubated on ice for 3 min, 300 uL of S.O.C. medium was added, and 100 µL was plated onto duplicates and incubated overnight at 37 °C. Agar plates were constructed with 1:1 (sterile d_I_H_2_O:agar) ratio, 500 µL Kanamycin antibiotics, and 2 mL of IPTG/xgal solution (Thermo Fisher Scientific, Wilmington, DE, USA). Single kanamycin-resistant colonies were selected based on their phenotypic color change (blue to white) and prepped for Colony PCR using One*Taq*^®^ Quick-Load^®^ 2× Master Mix with Standard Buffer (New England BioLabs) with a total reaction volume of 50 µL. Previous studies utilizing this in vitro system have revealed the formation of small, white satellite colonies not uncommon on agar plates with different reaction parameters. Extensive sequencing and analysis of these small, white satellite colonies has rendered no robust growth potential or sequencing data, rendering them insignificant in all reactions. PCR amplification was performed using 10 µM forward 5′–GCTTCCGGCTCGTATGTTGTGTGG–3′ and reverse primers 5′–GTTGGACGAGTCGGAATCGCAGA–3′ amplifying a 516 bp fragment. Initial denaturation of DNA template occurred at 94 °C for 2 min, cycle denaturation at 94 °C for 30 s, primer annealing at 60 °C for 1 min, and extension at 68 °C for 30 s for 35 cycles with a hold at 68 °C for 10 min. Samples were purified with QIAquick PCR Purification Kit silica spin columns (Qiagen, Hilden, Germany), loaded onto a 1% agarose gel for fragment amplification confirmation, and then sequenced in-house per manufacturer’s protocol for the SeqStudio Sequence Analyzer. Overall, each experiment was done in triplicate to strengthen the total white colony numbers. The first round of sequencing contained 20 white colonies from each experiment while the additional two repeats of each experiment had 22 white colonies sent out for sequencing (GeneWiz, South Plainfield, NJ, USA). The total number of white colonies for all Cas12a 20, 50, and 75 symmetrical homology arm experiments were brought to 64, 62, and 56 while Cas9 experiments (20, 50, and 75 symmetrical homology arm experiments) were brought to 61, 61, and 63 white colonies. Individual colony analysis was conducted using SnapGene4.2.11 software.

### 2.4. Statistical Analysis–Fisher’s Exact Test p < 0.05

Statistical analysis was performed using Fisher’s exact test which determines if two category variables contain nonrandom associations when the total number of samples is low. This statistical test was used in Table 1 and Table S1 which compared several experimental conditions between Cas12a and Cas9 as well as conditions within each category of RNP.

## 3. Results

### 3.1. Target Site in Genetic Readout System

**Figure 1:**
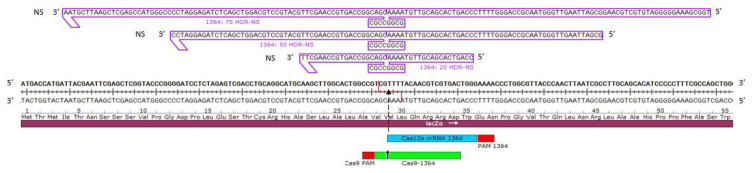
CRISPR/Cas12a/Cas9 target sites on the *LacZα* gene with various lengths of symmetrical HDR-NS oligonucleotides. The *lacZα* gene is shown with overlapping cleavage sites for CRISPR/Cas12a and CRISPR/Cas9. The staggered 5′, five base overhangs are generated by a Cas12a RNP and illustrated with a staggered red line while a Cas9 RNP generates a blunt ended cut shown by a dotted black arrow. Above the target sites are three oligonucleotides (denoted as the NS-strand in a 3′–5′ direction) with symmetrical homology arms of different lengths flanking an eight base *NOTI* site in the center.

Studies on fundamental mechanistic questions are best conducted in a reliable and robust model system. Thus, we chose the well-established *LacZα* gene in a well-known expression plasmid that has convenient and juxtaposed cut sites for Cas12a and Cas9 nucleases. The cut sites are located approximately at position 1364. Cas12a cleaves the target DNA two bases upstream from the 1364 site while Cas9 cleaves at the 1364 site. Guided by the consensus PAM sites, both nucleases cleave the plasmid template quickly and to completion [28]. Cleavage of dsDNA by Cas12a produces staggered ends while cleavage of dsDNA by Cas9 produces blunt ends. Figure 1 exhibits the target site and position of each respective programmable nuclease. Directly above the target sites are three donor DNA templates (ODNs) containing the full DNA sequence encased in purple with their polarities indicated. Each ODN was designed to contain 20, 50, or 75 bases of homology on each arm, while also providing for an eight base insertion of a *NOTI* restriction enzyme site. Thus, the donor DNA templates are 48, 108, and 158 bases total in length; all are constructed in the nonsense polarity (indicated by a 3′–5′ direction) and complementary to the sense strand (5′–3′ direction as indicated in Figure 1). Successful HDR would result in the precise insertion of the intact *NOTI* site with no secondary changes (precise HDR). Error-prone or imprecise HDR [28,33] is defined as a partial insertion of the eight base fragment or insertion with subsequent secondary mutagenesis surrounding the target site.

**Figure 2:**
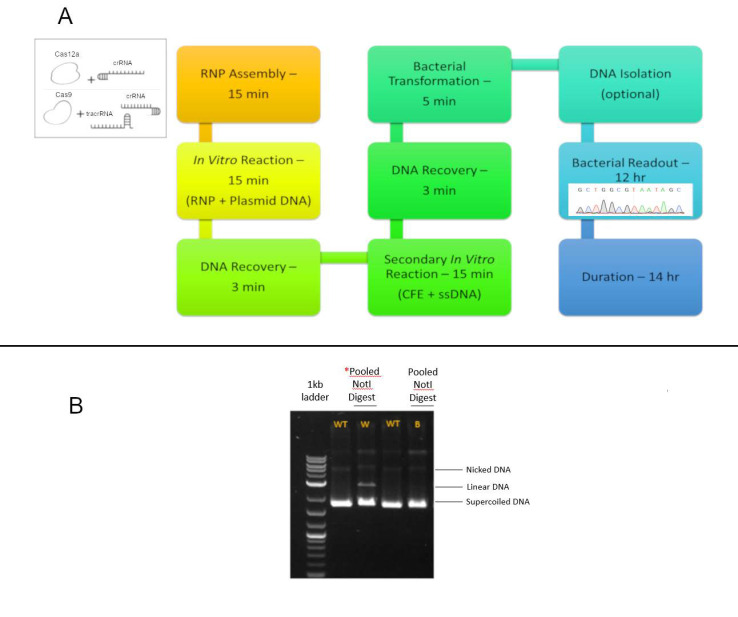
In vitro gene editing workflow and *NOTI* digestion assay. (**A**) In vitro workflow. The workflow starts with the complexation of CRISPR/Cas12 and/or CRISPR/Cas9 with the appropriate crRNA/tracrRNA+crRNA’s to form a ribonucleoprotein (RNP). After formation, the RNP is added to a reaction containing plasmid DNA, incubated for 15 min, and then cleaved DNA is recovered. The DNA is added to a second reaction with cell-free extract, ssDNA oligonucleotide, and undergoes DNA recovery once more. The recovered DNA is transformed into *Escherichia coli* bacteria, incubated overnight, and bacterial readout is observed after 12 h. (**B**) Pooled *NOTI* digestion assay. A *NOTI* digestion agarose gel is shown with five columns; the first column illustrates the 1 kb ladder and the remaining four columns show pooled white and blue colony samples along with a control, uncut *LacZ* plasmid. A linear band can be seen in the pooled white colony samples indicating several colonies with an intact *NOTI* site. The red asterisk signifies that only the white colony pool demonstrated *NOTI* cleavage. The pooled blue sample shows only wildtype (WT) uncut, plasmid DNA.

The in vitro, cell-free system used in these experiments has been previously established, through extensive control experimentation and replication to determine the optimal reaction parameters which prove the robustness and reproducibility of using this system [15,26,27,28]. Briefly (Figure 2A), the plasmid template is incubated with a CRISPR/Cas ribonucleoprotein (RNP) complex for 15 min to allow for complete cleavage of the *LacZα* gene containing plasmid molecules. The ssDNA donor fragment and the cell-free extract are subsequently added and incubated, after which the reaction mixture is transformed into bacteria and a visual readout is done after 12 h. Successful insertion of the *NOTI* site or any other genetic alteration can be observed by blue to white bacterial colony color change. This screening process enables a quick view to the success of the reaction, independent of precise or error-prone outcomes. Subsequent *NOTI* cleavage (Figure 2B) or DNA sequencing provides an accurate view of the population of genetically altered products. Blue colonies were sequenced to provide a control ensuring that the readout system continues to be validated by confirming that no genetic alterations have occurred.

#### 3.1.1. Precise HDR is Influenced by the Length of the Homology Arms of the Donor DNA Template in a Cas12a-Catalyzed Reaction

**Figure 3:**
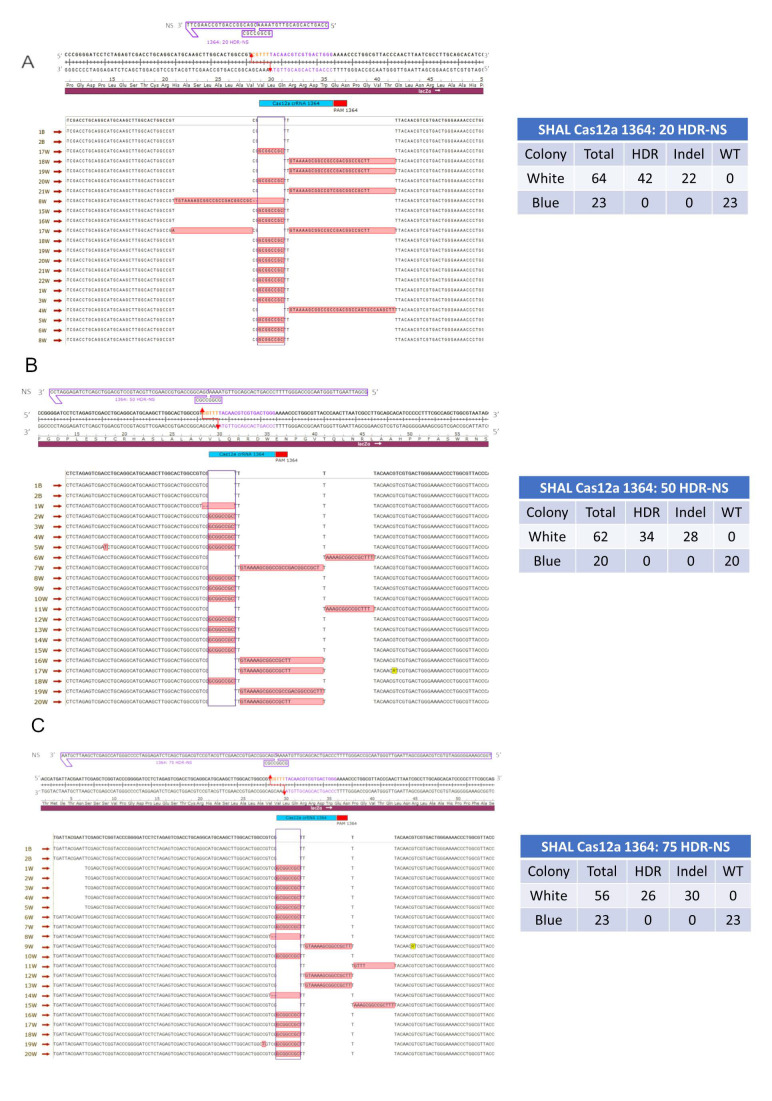
(**A**–**C**) Symmetrical homology arm lengths using CRISPR/Cas12a. Each image illustrates Cas12a in blue with the PAM site in red and a staggered red arrow indicating the five base, 5′ overhang created after cleavage occurs. (**A**–**C**) Symmetrical homology arm lengths of 20, 50, and 75 base pairs (48, 108, and 158 bases total). The nonsense symmetrical oligonucleotide with lengths of 20, 50, or 75 base pairs on either side of the cut site is located above the *lacZ* gene, contains an eight base *NOTI* site in the center, and is positioned in a 3′–5′ orientation. The purple box highlights the correct integration of a *NOTI* site while bases highlighted in red outside of the purple box, indicate unintended integration or deletion events. The table to the right of the images illustrates the total number of blue and white colonies sequenced and if they were found to harbor homology-directed repair, insertions or deletions (Indels), or a wild-type event. All blue colonies were found to harbor wild-type sequences represented by the two blue colonies (labeled 1B and 2B) in all images. All experiments were conducted in triplicate and the combined data is shown only in the tables present. The remaining sequencing data is shown in Figure S1 and the individual colony numbers for each trial are shown in Table S2.

The first combination of gene editing components tested included the Cas12a protein and a donor DNA fragment with 20 base homology arms upstream and downstream from the target site, which is represented by the eight base *NOTI* insertion fragment. Figure 3A provides a snapshot of the genetic diversity produced by this reaction, while the associated table displays the total number of blue and white colonies from the triplicates performed for this experiment. Out of 64 white colonies analyzed, 42 contained an exact insertion of the eight base fragment, while the remaining 22 colonies contained insertions ranging from 26 to 34 bases in length (see Figure S1 for additional sequences). This error-prone product likely involves NHEJ activity which, as previously demonstrated, occurs simultaneously in the in vitro reaction [15].

When the length of the donor DNA template was extended to 108 bases, with 50 base homology arms on either side of the target site, the number of precise HDR events decreased slightly to 34 precise HDR events (Figure 3B and Table S1). Therefore, the percentage of precise HDR products was found to be 55% while the 20 base homology arms experiment had approximately 66% precise HDR events. In this experimental condition, a somewhat diverse population of error-prone or imprecise gene editing products was observed. The 28 indel events observed in this experiment comprised insertions that ranged from 1 to 26 bases in length, a couple small base deletions, two 5 base insertions, and several instances of a base change in conjunction with an HDR event. The most frequent indel was a 16 bp insertion that occurred in 12 out of the 28 indel events.

Similar results were observed when the homology arms were increased to 75 bases on either side of the insertion site with approximately 47% of the gene edited plasmids bearing a precise insertion (Figure 3C). Here, the error-prone or imprecise genetic modifications fell into several categories. The indel categories were like those previously described; insertions ranged from 4 to 26 bases in length, several small base deletions, and several instances of single base change with an HDR event. Each homology arm length experiment with Cas12a was conducted in triplicate to bolster the white colony numbers for sequencing and analyzing purposes. At least two blue colonies were selected, as an internal control, sequenced, and analyzed with each experiment to ensure blue colonies remained unchanged, wildtype (WT) plasmid (Table S2).

#### 3.1.2. Precise HDR is Influenced by the Length of the Homology Arms of the Donor DNA Template in a Cas9-Catalyzed Reaction

**Figure 4:**
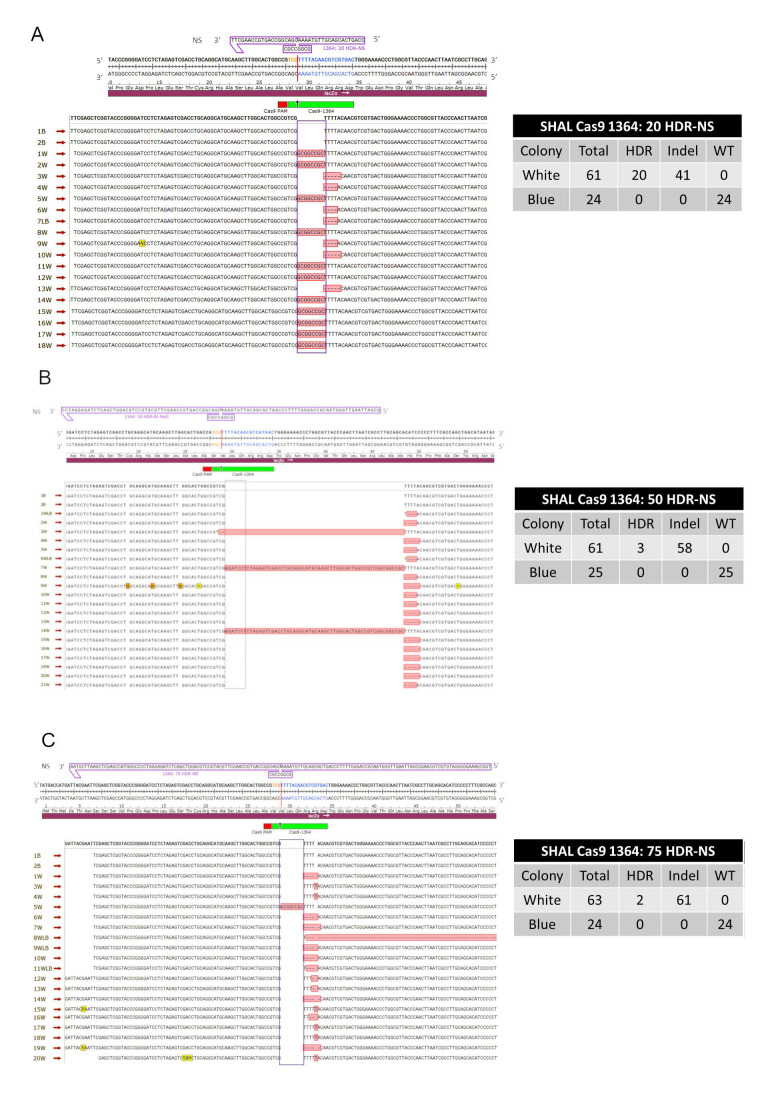
(**A**–**C**) Symmetrical homology arm lengths using CRISPR/Cas9. Each image illustrates the location of Cas9 in green and the PAM site in red. A solid red line indicates the blunt ended cleavage site. (**A**–**C**) symmetrical homology arm lengths of 20, 50, and 75 base pairs (48, 108, and 158 bases total). The nonsense symmetrical oligonucleotide with lengths of 20, 50, or 75 base pairs on either side of the cut site is located above the *lacZ* gene, contains an eight base *NOTI* site in the center, and is positioned in a 3′–5′ orientation. The purple box highlights the correct integration of a *NOTI* site and bases highlighted in red outside of the purple box indicate unintended integration or deletion events for each image. The table to the right of the images illustrates the total number of blue and white colonies sequenced and if they were found to harbor homology-directed repair, insertions or deletions (Indels), or a wild-type event. All blue colonies were found to harbor wild-type sequences represented by the two blue colonies (labeled 1B and 2B) in all images. All experiments were conducted in triplicate and the combined data is shown only in the tables present. The remaining sequencing data is shown in Figure S2 and the individual colony numbers for each trial are shown in Table S2.

We performed the same experimental length series using the same donor DNA templates described previously, however, the gene editing reactions were catalyzed by Cas9 cleavage (Figure 4A–C). Figure 4A–C demonstrates an overview of HDR and indel events; additional supportive data representing more colonies are provided in Figure S2 and Table S2 shows the number of colonies found in each trial for all homology arm lengths. Interestingly, a robust level of precise HDR insertion was now observed only with the DNA donor template bearing the shortest degree of homology arms. With a DNA template bearing 20 bases of homology on either side of the designated insertion fragment, approximately 33% of the gene edited colonies, appearing white in the genetic readout system, contained only the eight base insertion fragment. In contrast, as the length of homology arms increased to 50 and to 75, only five gene edited colonies (out of 124 colonies) contained a precise insertion. Once again, a diverse population of error-prone or imprecise indels were observed with each of the donor templates, and within these three experiments, the populations were different. A majority of the indel populations consisted of small deletions ranging from 2 to 5 bases, several instances of a single base insertion, and a couple insertions greater than 40 bases in length. Among these three experimental conditions there were a total of 160 indel events and 25 precise HDR events.

As mentioned above, previous studies have indicated the importance of the CFE component in gene editing reactions [27]. Once this data for both Cas12a and Cas9, in combination with all three homology arm lengths, was established, select experiments with and without the addition of CFE were conducted. The results show the CFE continues to be a vital component of the in vitro system by providing the enzymatic activity to achieve gene edited outcomes (Figure S3A–B). Both experiments demonstrated that excluding CFE yielded only blue colonies whereas reactions with CFE yielded both blue and white colonies, indicative of gene editing activity occurring. Previous publications using the in vitro system, have also shown extensive experimentation with several controls including but not limited to, RNP amount, CFE concentration, oligonucleotide template strand bias etc., to establish this robust and reproducible system [15,26,27,28]. In addition, to the sequencing data shown (Figure 3 and Figure 4), we compared next-generation sequencing (NGS) to direct sanger sequencing using analysis programs TIDES [34] and CRIS.py [35] to exemplify that the results were not an outcome of repair occurring in *E. coli* after transformation. We found NGS to have similar HDR and indels levels compared to sanger sequencing (Figure S4), eliminating the possibility of post-transformation repair occurring in *E. coli.*

**Figure 5:**
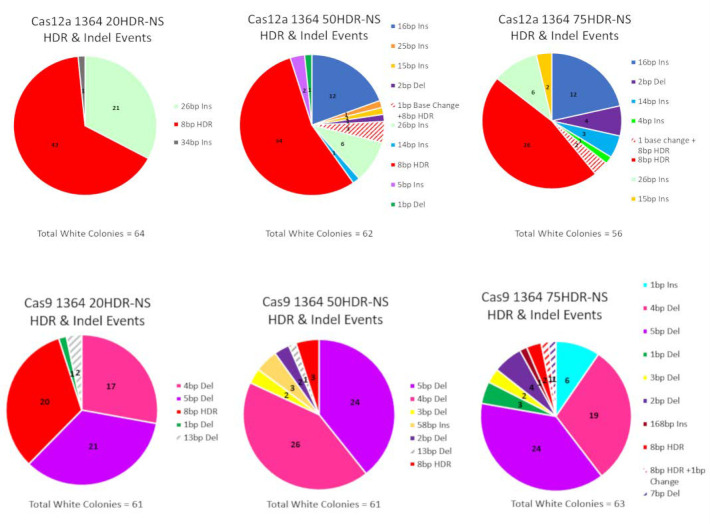
Frequency of HDR and Indel events in CRISPR/Cas12a and CRISPR/Cas9 reactions. Each pie chart represents a reaction condition utilizing CRISPR/Cas12a or CRISPR/Cas9 in combination with the 20, 50, or 75 base homology arm ODN and the triplicate experiments done within each nuclease. The total number of events (HDR or Indels) and the frequency of those events are illustrated by different colors. A precise integration of the eight base *NOTI* site is depicted in red and the remaining colors depict various types of Indels.

A summary profile of products from all white colonies in the CRISPR/Cas reactions based on the length of the ssDNA donor template are provided in Figure 5. The top row features products created in Cas12a-driven reactions along with the indel populations consisting primarily of large insertions and a smaller number of deletions. In contrast, reactions catalyzed by Cas9 produced a few insertions yet a majority of indels revealed a consistent pattern of small deletions ranging from 1 to 5 base pairs. In general, the length of the donor DNA template does not appear to alter the overall profile. This indicates that the processing of the break site may be independent of the length of the donor template used to repair it.

A statistical comparison between Cas12a- and Cas9-catalyzed reactions was conducted and is provided by Table 1. We utilized Fisher’s exact test to determine the statistical significance of the programmable nuclease and donor DNA template combinations. Cas12a reactions with all three homology arm lengths were found to be statistically significant compared to all Cas9-driven reactions. Cas12a dominated in each experiment having at least twice as many HDR events as Cas9. Between these experiments Cas9 contained 25 precise HDR events while Cas12a exhibited 102 precise HDR events. The differences between these combinations were found to be statistically significant with relevant *p*-values shown (Table 1). Internal combinatorial comparisons were also conducted revealing the Cas12a 20 base homology arms experiment to be significant in comparison only to the reaction with 75 base homology arms. While, Cas9 in conjunction with the 20 base homology arms was found to be statistically significant when compared to the two other homology arms lengths in Cas9 reactions; these analyses are presented in Table S1.

## 4. Discussion

As the CRISPR/Cas gene editing technology continues to dominate the genetic engineering of mammalian cells, efforts are underway to elucidate the reaction parameters that surround its precision and efficiency. Previous studies have focused on the degree of homology between the CRISPR guide RNA and the target site [5,28,31] and the specificities of the CRISPR/Cas complex [17,36]. Several studies have provided valuable information on the influence that ssDNA donor templates can have on the efficiency of HDR [8,16,17,22,23,24,25]. While these informative results have guided the overall design of the donor templates, cell-based systems rely on the consistent performance of reaction parameters some of which are neither consistent nor controllable. First, the transfection efficiency of biomaterial into different cellular lines is notoriously variable. Second, there is no evidence to support that equal levels of the donor template reach the nucleus; entry is likely blocked at the nuclear membrane. Therefore, both false positive and false negative outcomes can be skewed by the number of donor templates present at the target site. And, third, the repair efficiencies among cell lines, and even within passage numbers of these cell lines, could be different thus the repair of CRISPR/Cas induced double-strand cleavage can also be highly variable.

We created a reaction environment where some of these variables can be eliminated to enable studies seeking to comprehend the influence of ssDNA donor templates on the process of HDR. By using a robust and well-validated cell-free extract (CFE) in vitro system [26,27], we can now report with confidence that the length of the donor template, in fact, does determine the level of productive and more importantly, nonproductive, gene editing. While the CFE system has drawbacks, it provides us the opportunity to visualize all genetic outcomes of the gene editing events in a reproducible and quantifiable fashion. Due to the simplicity and straightforwardness of this reaction, gene editing events can be evaluated within 12 h of reaction completion.

Our results indicate that homology arms of 20 bases are optimal, however, homology arms of 50 and 75 bases would also be sufficient in CRISPR/Cas12a-directed HDR reactions and that the indel population consists mostly of insertions ranging from 12 to 34 base pairs (with a high frequency of a 26 base pair fragment), all downstream from the target site. Precise HDR events in CRISPR/Cas9-driven reactions appear most readily when the donor template contains 20 base homology arms, contrasting sharply, in a statistically significant way, only when compared to the results obtained with the 50 and 75 length ssDNA templates (Table S1). For Cas12a and Cas9 reactions, a total of 182 and 185 white colonies were observed when all positive results were combined from all homology arm length experiments; thus demonstrating, that each CRISPR/Cas complex is active at a similar level in the CFE system. Yet, precise HDR was found to be more prevalent in Cas12a reactions (56%) in comparison to reactions where Cas9 executed the DSB’s (13.5%). These results are not surprising and, in fact, are consistent with previously published data suggesting that a higher percentage of precise HDR activity is observed when Cas12a catalyzes the DSB [15]. We suspect that these outcomes are likely due to the unique cleavage patterns created by the individual nucleases.

For Cas12a, staggered five base overhangs can activate single-stranded DNA annealing (SSA) protein activity providing suitable activity for stable homologous alignment. SSA [6,37,38,39] is well-established as a pathway in which complementary ssDNA molecules pair to create a duplex structure that can bridge dsDNA quite efficiently for generating insertions and genetic disruption in mammalian cells. While this pathway is often viewed as mutagenic in nature, small specific insertions designed to be incorporated at the exact target site can be obtained. In contrast, Cas9 cleavage leaves blunt ends which are usually less genetically recombinant because they lack single-stranded character and, thus, do not enable high levels of precise HDR naturally.

An interesting observation is reflected in the population of indels that either accompany or form independently of HDR. When they accompany HDR, the process is known as error-prone HDR, originally shown in zebrafish [33] and more recently observed in both mammalian cells [5] and cell-free extracts [28]. Clearly, the staggered ends created by Cas12a cleavage provide an attractive template for resection activity catalyzed by members of the NHEJ pathway. As a result, long stretches of ssDNA are generated which can enable better homologous alignment. Additionally, as such, error-prone HDR reflected by large insertions would be more prevalent in Cas12a-driven reactions; our data support this notion.

A striking result found in this data was the amount of error-prone HDR activity seen in the Cas12a reaction with the donor DNA template of 20 base homology arms. A unique DNA fragment was found in a majority of error-prone outcomes, a 26 base insertion, at the exact same site, occurring in 21 of the 64 gene editing events. The inserted sequence is derived from the donor template. In the original work, establishing the CRISPR/Cas12a catalyzed reaction in a cell-free extract system, short fragment insertion was among the most prominent outcomes, and is likely being recapitulated here [15,26,27].

Contrary to this, small indels can be predicted in reactions where NHEJ activity must engage resection activity of blunt ends, a less favorable template, and a result observed from Cas9 cleavage in this study. Overall, these data provide insight into the mechanisms of insertion in gene editing reactions where Cas9 serves as the nuclease. By leaving blunt ends, the termini are more reactive when short fragments are provided in the reaction mixture. This is due to the amount of time that is taken up by searching for homology which is reduced substantially when the quantity of DNA being scanned is small.

Our data provide some insight into the importance of homology arm length and the effect it can have on gene editing. Although many cell-based assays utilize Cas9 to execute the double strand break, the longer the homology arms of the donor are, the higher levels of homology directed repair are observed. In our experiments using Cas9, we see a decrease in the efficiency of precise repair. We believe that short donor fragments with homology arms of 50 bases or less likely follow a different pathway than their counterparts bearing homology arms of 100–200 bases and higher. These longer donor templates likely initiate the repair of the broken site via a pathway more akin to homologous recombination, and one used in the process of gene knock-in. In cases where the donor DNA length is restricted, it is likely that single-stranded dependent strand annealing (SDSA), exemplified by the process known as ExACT is followed [13,16,18]. We conclude that the length of the donor DNA template may dictate the pathway of repair or replacement followed as a cell re-engineers the broken chromosomal site.

Our results provide foundational information about the subtleties that have a major impact on the efficiency and efficacy of the overall gene editing reaction. Modest changes in the length of DNA homology appear to influence the molecular mechanisms of gene repair, directing the pathway that is used to both regulate and execute DNA modification.

## Figures and Tables

**Table 1:** Statistical comparison between CRISPR/Cas12a and CRISPR/Cas9 using homology arm lengths of 20, 50, and 75 bases. Fisher’s exact test was used to compare Cas12a with Cas9 using three different lengths of symmetrical homology arms. The total number of precise HDR events and Indels are presented along with the column totals and *p*-values < 0.05 for the three comparisons. *p*-values noted with * indicate statistically significant data within that comparison.

* Cas12a vs. Cas9-20HDR-NS				
	HDR	Indel	Total	
Cas12a 1364 20HDR-NS	42	22	64	
Cas9 1364 20HDR-NS	20	41	61	
Marginal column totals	62	63	125	
*p*-value < 0.05				0.0003 *
* Cas12a vs. Cas9 50HDR-NS				
	HDR	Indel	Total	
Cas12a 1364 50HDR-NS	34	28	62	
Cas9 1364 50HDR-NS	3	58	61	
Marginal column totals	37	86	123	
*p*-value < 0.05				0.0001 *
* Cas12a vs. Cas9-75HDR-NS				
	HDR	Indel	Total	
Cas12a 1364 75HDR-NS	26	30	56	
Cas9 1364 75HDR-NS	2	61	63	
Marginal column totals	28	91	119	
*p*-value < 0.05				0.00001 *

## Supplementary Materials

The following are available online at https://www.mdpi.com/2073-4425/11/10/1160/s1, Figure S1A–C: Additional data for Figure 3A–C—symmetrical homology arm lengths using CRISPR/Cas12a, Figure S2A–C: Additional data for Figure 4A–C—symmetrical homology arm lengths using CRISPR/Cas9, Figure S3A,B: CRISPR/Cas control with and without cell-free extract, Figure S4A,B: Comparison of direct in vitro product sequencing via Sanger and next-generation sequencing, Table S1: Fisher’s exact test comparing 20, 50, and 75 base homology arms within CRISPR/Cas12a and within CRISPR/Cas9, and Table S2: CRISPR/Cas12a and CRISPR/Cas9 20, 50, and 75 homology arm length triplicate comparison within each nuclease.
