# Supplementary material for: The Diversity of Genetic Outcomes from CRISPR/Cas Gene Editing is Regulated by the Length of the Symmetrical Donor DNA Template
Source: Genes (Basel). 2020 Sep 30;11(10):1160. doi: 10.3390/genes11101160 (PMC7599521; doi:10.3390/genes11101160)
Supplement: Supplementary file 1 [file genes-11-01160-s001.pdf]

A

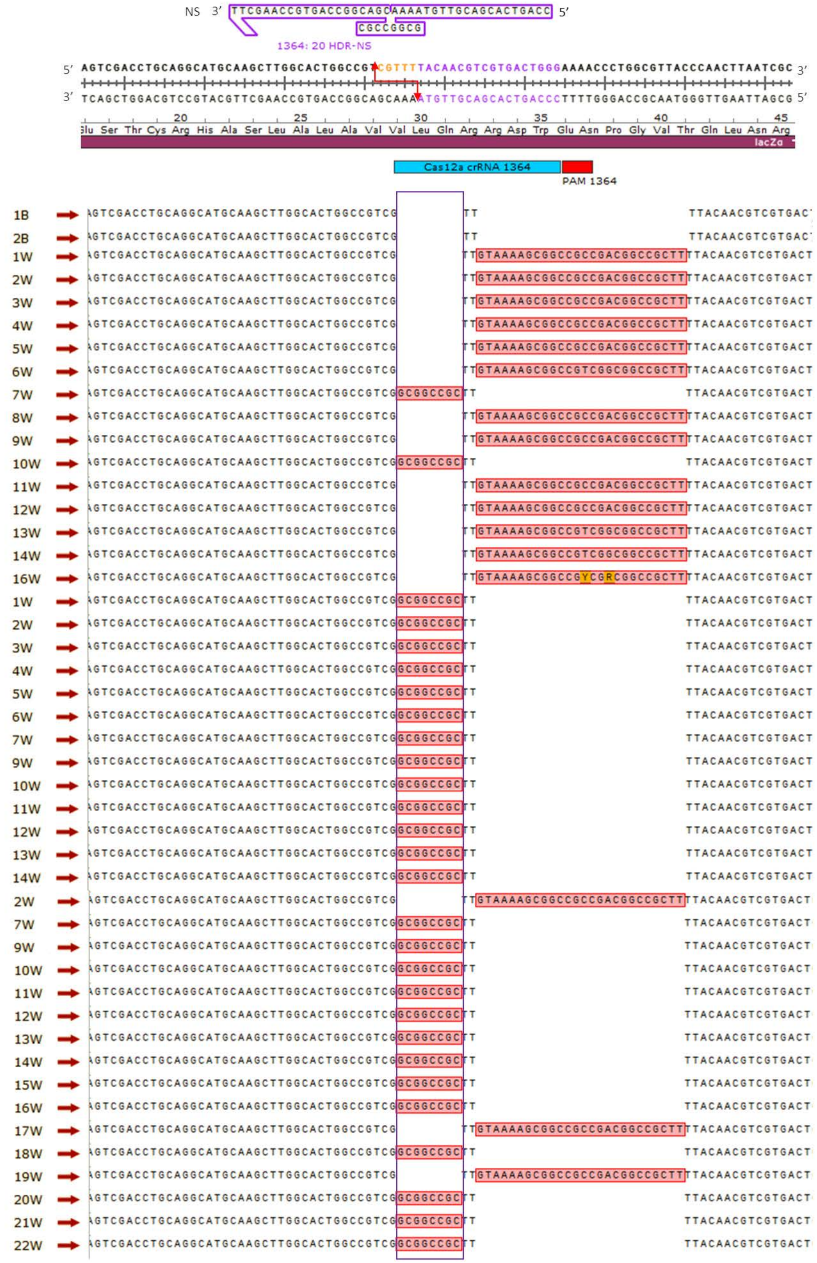

NS 3' TTCGAACCGTGACCGGCAGCAAAATGTTGCAGCACTGACC 5'
CGCCGGCG
1364: 20 HDR-NS
5' AGTCGACCTGCAGGCATGCAAGCTTGGCACTGGCCGTCGTTTTACAACGTCGTGACTGGGAAAACCCTGGCGTTACCCAACTTAATCGC 3'
3' TCAGCTGGACGTCCGTACGTTCGAACCGTGACCGGCAGCAAAATGTTGCAGCACTGACCCTTTTGGGACCGCAATGGGTTGAATTAGCG 5'
20
25
30
35
40
45
Glu Ser Thr Cys Arg His Ala Ser Leu Ala Leu Ala Val Val Leu Gln Arg Arg Asp Trp Glu Asn Pro Gly Val Thr Gln Leu Asn Arg
lacZα
Cas12a crRNA 1364
PAM 1364
1B
2B
1W
2W
3W
4W
5W
6W
7W
8W
9W
10W
11W
12W
13W
14W
16W
1W
2W
3W
4W
5W
6W
7W
9W
10W
11W
12W
13W
14W
2W
7W
9W
10W
11W
12W
13W
14W
15W
16W
17W
18W
19W
20W
21W
22W

B

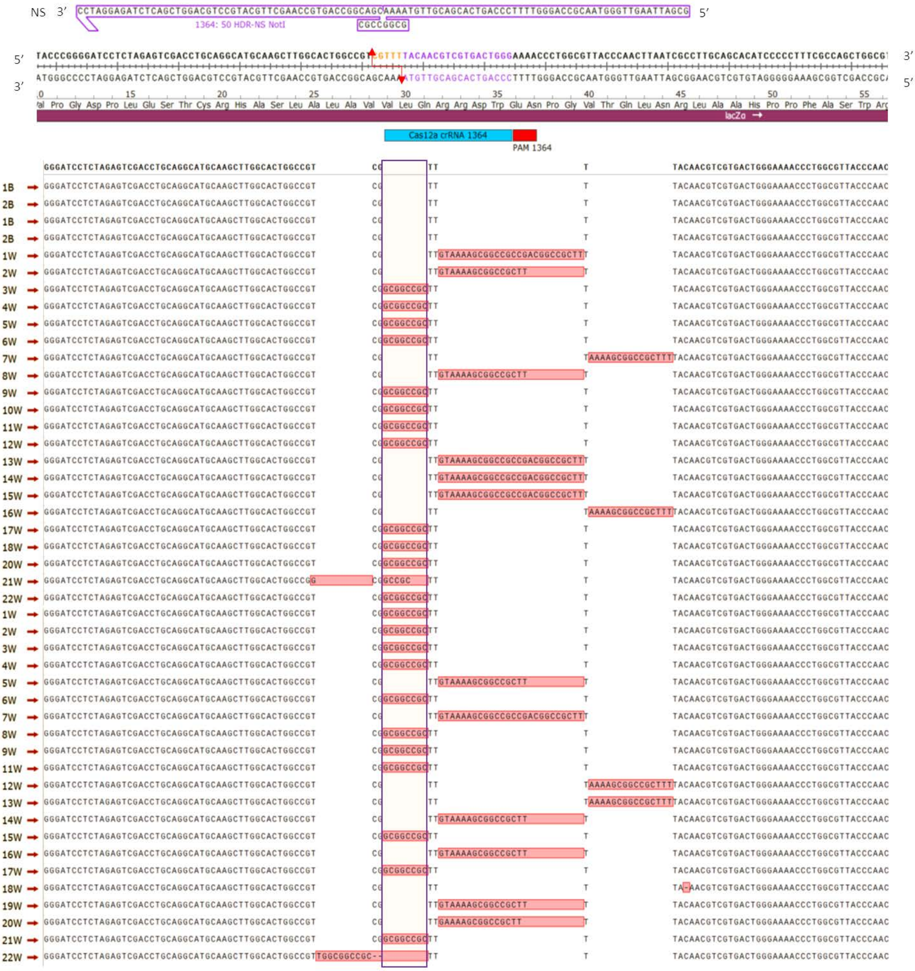
NS
1364: 50 HDR-NS NotI
CGCCGGCG
lacZα
Cas12a crRNA 1364
PAM 1364

C

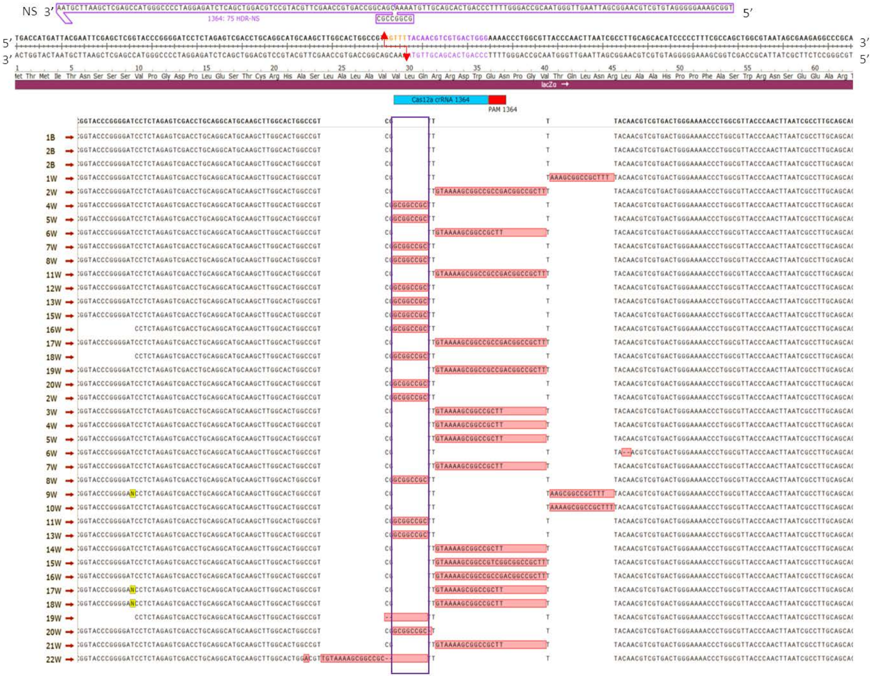
NS 3′
5′
1364: 75 HDR-NS
CGCCGGCG
5′
3′
3′
5′
lacZα
Cas12a crRNA 1364
PAM 1364
1B
2B
2B
1W
2W
4W
5W
6W
7W
8W
11W
12W
13W
15W
16W
17W
18W
19W
20W
2W
3W
4W
5W
6W
7W
8W
9W
10W
11W
13W
14W
15W
16W
17W
18W
19W
20W
21W
22W

Figure S1A-C: Additional data for Figure 3A-C - Symmetrical Homology Arm Lengths using CRISPR/Cas12a. Each image illustrates Cas12a in blue with the PAM site in red and a staggered red arrow indicating the 5 base, 5′ overhang created after cleavage occurs. **(A-C) Additional data for Symmetrical Homology Arm Length of 20, 50, and 75 base pairs (48, 108, and 158 bases total).** The nonsense symmetrical oligonucleotide with lengths of 20, 50, or 75 base pairs on either side of the cut site is located above the *lacZ* gene, contains an eight base *NOT*I site in the center, and is positioned in a 3′ to 5′ orientation. The purple box highlights the correct integration of a *NOT*I site while bases highlighted in red outside of the purple box, indicate unintended integration or deletion events. The total number of colonies and the category for each event can be found in the tables located in Figure 3A-C. All blue colonies were found to harbor wild-type sequences and are represented by several blue colonies (labeled 1B and 2B) in all images.

A

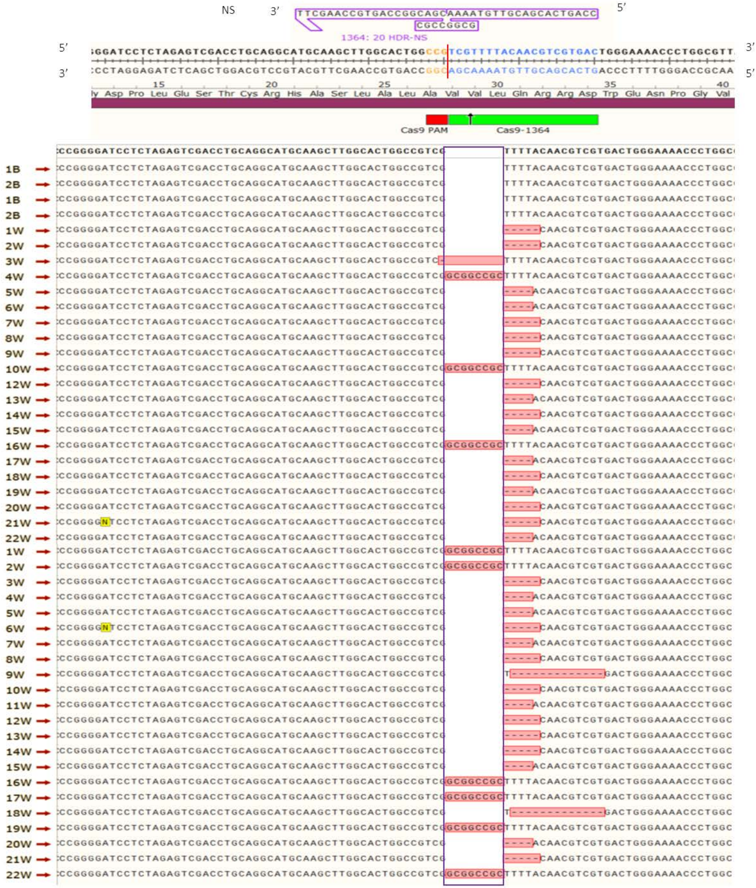

NS
3'
TTCGAACCGTGACCGGCAGCAAAATGTTGCAGCACTGACC
5'
CGCCGGCG
1364: 20 HDR-NS
5'
3'
Cas9 PAM
Cas9-1364

B

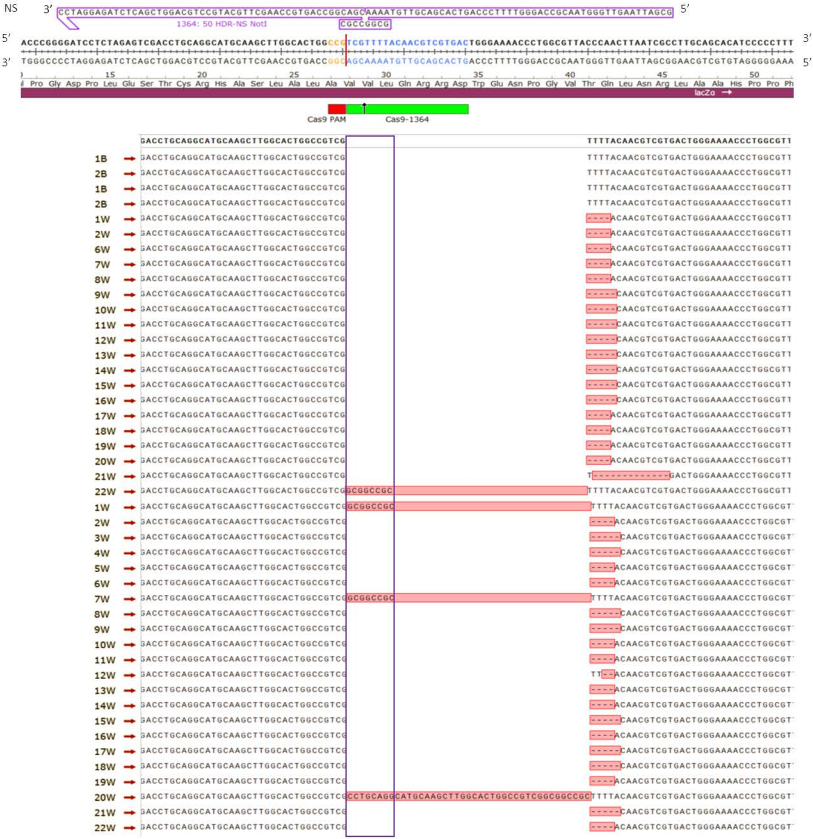
NS
3′
5′
1364: 50 HDR-NS NotI
CGCCGGCG
5′
3′
3′
5′
lacZα
Cas9 PAM
Cas9-1364
1B
2B
1B
2B
1W
2W
6W
7W
8W
9W
10W
11W
12W
13W
14W
15W
16W
17W
18W
19W
20W
21W
22W
1W
2W
3W
4W
5W
6W
7W
8W
9W
10W
11W
12W
13W
14W
15W
16W
17W
18W
19W
20W
21W
22W

C

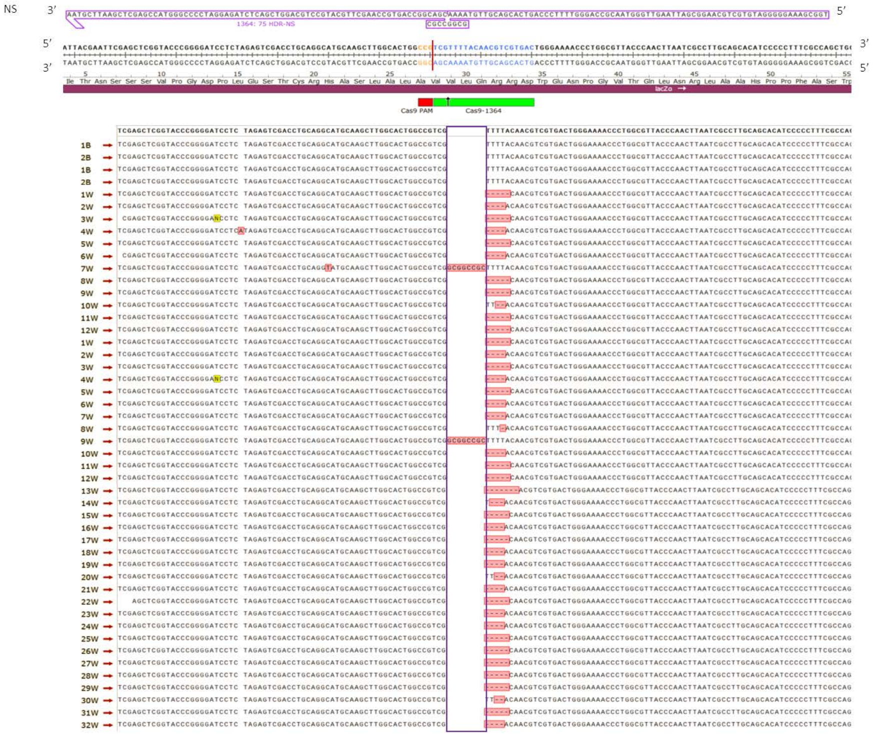
NS
1364: 75 HDR-NS
CGCCGGCG
Cas9 PAM
Cas9-1364
lacZα
1B
2B
1B
2B
1W
2W
3W
4W
5W
6W
7W
8W
9W
10W
11W
12W
1W
2W
3W
4W
5W
6W
7W
8W
9W
10W
11W
12W
13W
14W
15W
16W
17W
18W
19W
20W
21W
22W
23W
24W
25W
26W
27W
28W
29W
30W
31W
32W

Figure S2A-C: Additional data for Figure 4. A-C - Symmetrical Homology Arm Lengths using CRISPR/Cas9. Symmetrical Homology Arm Lengths using CRISPR/Cas9. Each image illustrates the location of Cas9 in green and the PAM site in red. A solid red line indicates the blunt ended cleavage site. **(A-C) Symmetrical Homology Arm Lengths of 20, 50, and 75 base pairs (48, 108, and 158 bases total).** The nonsense symmetrical oligonucleotide with lengths of 20, 50, or 75 base pairs on either side of the cut site is located above the *lacZ* gene, contains an eight base *NOT*I site in the center, and is positioned in a 3′ to 5′ orientation. The purple box highlights the correct integration of a *NOT*I site and bases highlighted in red outside of the purple box indicate unintended integration or deletion events for each image. The total number of colonies and the category for each event can be found in the tables located in Figure 4A-C. All blue colonies were found to harbor wild-type sequences and are represented by the four blue colonies (labeled 1B and 2B) in all images.

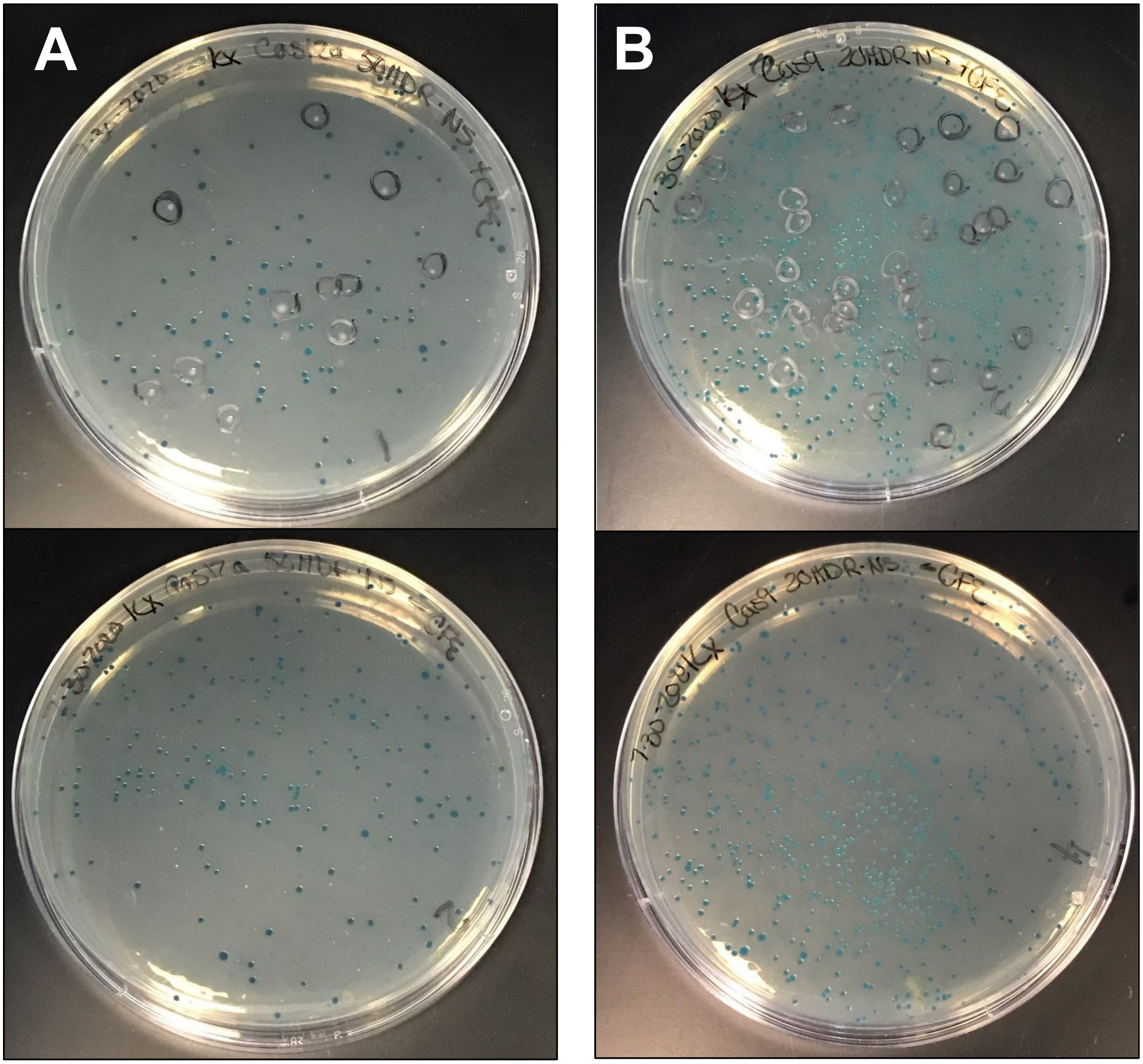

Figure S3A-B: CRISPR/Cas control reactions with and without cell-free extract. A) Cas12a 1364 reaction with 50 base homology arm lengths. B) Cas9 1364 reaction with 20 base homology arm lengths. The left image contains two Cas12a plates and the right image contains two Cas9 plates. The top plates for each encompass reactions with cell-free extract while the bottom plate reactions do not contain cell-free extract. The top plates for both Cas12a and Cas9 display blue and white colonies; (all white colonies are circled in black) and only blue colonies are present on the bottom plates.

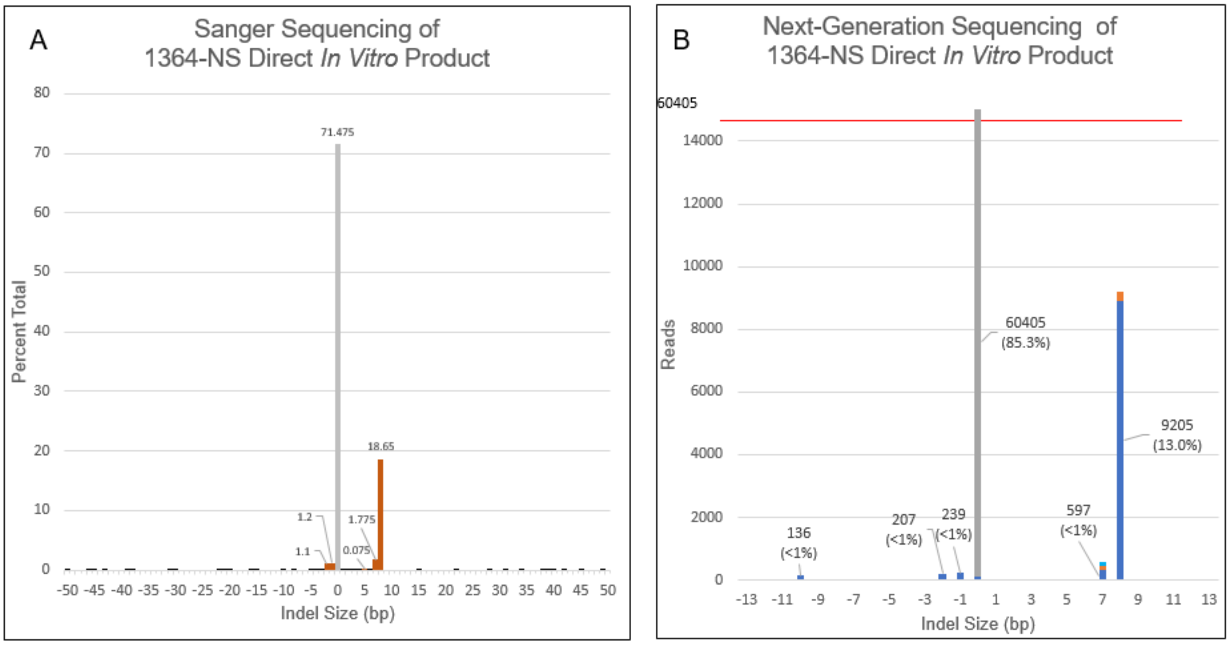

Figure S4A-B: Comparison of Direct *In Vitro* Product Sequencing via Sanger and Next-Generation Sequencing. For both figures, DNA was isolated and PCR-amplified directly after recircularization, with no bacterial transformation. **A)** Amplicons were sequenced via Sanger sequencing, and resultant indels were deconvoluted using TIDE. The grey bar at 0 represents wild-type, perfectly repaired sequences. Orange bars represent statistically significant indels, as determined by pearson's r via TIDE. Black bars represent non-significant background. **B)** Amplicons were sequenced via Illumina TruSeq next-generation sequencing, and resultant indels were determined using CRIS.py. Each bar consists of stacked sequences containing all sequence variations of the appropriate indel length in blue, orange, and cyan (in order of contribution percentage). The grey bar at 0 represents wild-type sequences. The horizontal orange bar represents an axis break from 14,000 to 60,405 reads.

Table S1: Fisher's exact test comparing 20, 50, and 75 base homology arms within CRISPR/Cas12a and within CRISPR/Cas9.

Cas12a 20HDR-NS vs. Cas12a 50HDR-NS					*Cas9 20HDR-NS vs. Cas9 50HDR-NS				
	HDR	Indel	Total			HDR	Indel	Total	
Cas12a 1364 20HDR-NS	42	22	64		Cas9 1364 20HDR-NS	20	41	61	
Cas12a 1364 50HDR-NS	34	28	62		Cas9 1364 50HDR-NS	3	58	61	
Column Totals	76	50	126		Column Totals	23	99	122	
P-Value <0.05				0.2748	P-Value <0.05				0.0001
*Cas12a 20HDR-NS vs. Cas12a 75HDR-NS					*Cas9 20HDR-NS vs. Cas9 75HDR-NS				
	HDR	Indel	Total			HDR	Indel	Total	
Cas12a 1364 20HDR-NS	42	22	64		Cas9 1364 20HDR-NS	20	41	61	
Cas12a 1364 75HDR-NS	26	30	56		Cas9 1364 75HDR-NS	2	61	63	
Column Totals	68	52	120		Column Total	22	102	124	
P-Value <0.05				0.0428	P-Value <0.05				0
Cas12a 50HDR-NS vs. Cas12a 75HDR-NS					Cas9 50HDR-NS vs. Cas9 75HDR-NS				
	HDR	Indel	Total			HDR	Indel	Total	
Cas12a 1364 50HDR-NS	34	28	62		Cas9 1364 50HDR-NS	3	58	61	
Cas12a 1364 75HDR-NS	26	30	56		Cas9 1364 75HDR-NS	2	61	63	
Column Totals	60	58	118		Column Totals	5	119	124	
P-Value <0.05				0.461	P-Value <0.05				0.6772

Table S1: Fisher's exact test comparing 20, 50, and 75 base homology arms within CRISPR/Cas12a and within CRISPR/Cas9. Fisher's exact test was used to compare the three lengths of homology arms within each nuclease. Statistically significant conditions within the comparisons are highlighted in red along with the associated P-value. The total number of precise HDR events and Indels are indicated along with the column totals. The P-value was set to < 0.05 for all comparisons.

Table S2: CRISPR/Cas12a and CRISPR/Cas9 20, 50, and 75 homology arm length triplicate comparison within each nuclease.

Cas12a 1364 20HDR-NS						Cas9 1364 20HDR-NS					
**Trial**	**Colony Color**	**Total**	**HDR**	**Indel**	**WT**	**Trial**	**Colony Color**	**Total**	**HDR**	**Indel**	**WT**
1	White	20	4	16	0	1	White	18	11	7	0
	Blue	19	0	0	19		Blue	20	0	0	20
2	White	22	20	2	0	2	White	21	3	18	0
	Blue	2	0	0	2		Blue	2	0	0	2
3	White	22	18	4	0	3	White	22	6	16	0
	Blue	2	0	0	2		Blue	2	0	0	2
Cas12a 1364 50HDR-NS						Cas9 1364 50HDR-NS					
**Trial**	**Colony Color**	**Total**	**HDR**	**Indel**	**WT**	**Trial**	**Colony Color**	**Total**	**HDR**	**Indel**	**WT**
1	White	20	11	9	0	1	White	20	0	20	0
	Blue	16	0	0	16		Blue	21	0	0	21
2	White	21	12	9	0	2	White	19	1	18	0
	Blue	2	0	0	2		Blue	2	0	0	2
3	White	21	11	10	0	3	White	22	2	20	0
	Blue	2	0	0	2		Blue	2	0	0	2
Cas12a 1364 75HDR-NS						Cas9 1364 75HDR-NS					
**Trial**	**Colony Color**	**Total**	**HDR**	**Indel**	**WT**	**Trial**	**Colony Color**	**Total**	**HDR**	**Indel**	**WT**
1	White	20	10	10	0	1	White	19	1	18	0
	Blue	20	0	0	20		Blue	20	0	0	20
2	White	16	10	6	0	2	White	12	0	12	0
	Blue	2	0	0	2		Blue	2	0	0	2
3	White	20	6	14	0	3	White	32	1	31	0
	Blue	2	0	0	2		Blue	2	0	0	2

Table S2: CRISPR/Cas12a and CRISPR/Cas9 20, 50, and 75 homology arm length triplicate comparison within each nuclease. The table illustrates the total number of blue and white colonies sequenced for each homology arm length experiment (20, 50, or 75) and the triplicates performed within each experiment conducted for Cas12a (left) and Cas9 (right). The table displays which colonies were found to harbor homology-directed repair, insertions or deletions (Indels), or wild-type events. All blue colonies were found to harbor wild-type sequences. Each homology arm length shows comparable numbers of both blue and white colonies for all experiments within each nuclease.
